# Variable-Focus Liquid Lens Integrated with a Planar Electromagnetic Actuator

**DOI:** 10.3390/mi7100190

**Published:** 2016-10-17

**Authors:** Liang Wang, Junping Duan, Binzhen Zhang, Wanjun Wang

**Affiliations:** 1 Science and Technology on Electronic Test & Measurement Laboratory, North University of China, Taiyuan 030051, China; biomemswl@gmail.com (L.W.); duanjunping@nuc.edu.cn (J.D.); 2 School of Instrument and Electronics, North University of China, Taiyuan 030051, China; 3 Department of Mechanical Engineering, Louisiana State University, Baton Rouge, LA 70803, USA; wang@me.lsu.edu

**Keywords:** liquid lens, focal length, planar coil, ultraviolet photolithography (UV-LIGA), electromagnetic actuation

## Abstract

In this paper, we design, fabricate and characterize a new electromagnetically actuated variable-focus liquid lens which consists of two polymethyl methacrylate (PMMA) substrates, a SU-8 substrate, a polydimethylsiloxane (PDMS) membrane, a permanent magnet and a planar electromagnetic actuator. The performance of this liquid lens is tested from four aspects including surface profiling, optical observation, variation of focal length and dynamic response speed. The results shows that with increasing current, the optical chamber PDMS membrane bulges up into a shape with a smaller radius of curvature, and the picture recorded by a charge-coupled device (CCD) camera through the liquid lens also gradually becomes blurred. As the current changes from −1 to 1.2 A, the whole measured focal length of the proposed liquid lens ranges from −133 to −390 mm and from 389 to 61 mm. Then a 0.8 A square-wave current is applied to the electrode, and the actuation time and relaxation time are 340 and 460 ms, respectively. The liquid lens proposed in the paper is easily integrated with microfluidic chips and medical detecting instruments due to its planar structure.

## 1. Introduction

The variable-focus liquid microlens plays an important role in modern micro-optics systems, optical communication systems and biomedical detection systems, such as intraoral cameras or skin measurement devices [1,2,3]. Different from traditional lenses, which vary their focal length by relying on adjusting the relative positions of the lenses and sensors through conventional mechanical devices, variable-focus liquid lenses generally change their own refractive index or droplet curvature to achieve zooming purposes. The variable-focus liquid lens has many advantages, including a simple structure, low energy consumption, etc. [4,5,6,7,8,9,10]. 

As the market of consumer electronics blooming, the demand for variable-focus lenses is increasing. Many new types of variable-focus liquid lenses have been reported. The actuating mechanism of the reported variable-focus liquid lenses can be classified into four major types: electromagnetic actuation [11], the refraction effect of liquid crystal [12,13,14], electrowetting on dielectric [15,16,17], and the dielectric electrophoresis effect [18,19,20]. Each mechanism has its own advantages and disadvantages. The liquid crystal–type lenses have the advantages of fast response and high sensitivity. However, most of them need a polarizer because they are polarization dependent, and they will obtain blurred images if the temperature is below the liquid crystal’s transition temperature. Electrowetting liquid lenses have a relatively larger tunable range, but they usually require a very high applied voltage (usually more than 80 V). Furthermore, they are difficult to apply to portable equipment. Dielectric electrophoresis effect–type lenses adopt two non-conductive liquids, operate at small volumes, avoid electrolysis owing to the negligible current in the liquids, and have the merits of accurate regulation, quick response time and low power consumption. However, similar to the electrowetting liquid lenses, most dielectric electrophoresis lenses need a high voltage, which limits their further development. Compared with these methods, the liquid lenses driven by electromagnetic actuation are the most attractive because of their simple actuating and zooming principle, wide zoom range, fast response, and low cost. However, in a conventional electromagnetically actuated liquid lens, to deform the optical chamber membrane, an external auxiliary coil is used to provide the driven force. Yu et al. [21] reported a tunable electromagnetically actuated liquid-filled lens; this liquid lens has a simple configuration and a wide tunable range of focal length, but due to the use of an external electromagnet, this liquid lens has a large volume which make it difficult to install in a plane or realize the integration of the lens body and actuating components. As a result, its applications in biomedical detection and micro-optics systems are severely limited.

The planar electromagnetic actuator, especially that made by micro-fabrication technology, has the advantages of small volume and suitability for planar integration. In recent years, the method of integrating a planar coil with a permanent magnet as a micro-actuator has been widely used in micropumps, microvalves and other microelectromechanical systems (MEMS) devices because they can obtain large displacements and a big driving force in the case of a low applied voltage [22,23,24]. Tilmans et al. [22] introduced a fully integrated electromagnetic microrelay. They used a multilayer Cu coil to actuate a movable NiFe armature for closing Au contacts. Typical properties of this relay include: a response time of 1 ms, a driving voltage of 2 V, and a service life under low-level load of about 107 cycles. Hartley et al. [24] reported a large-throw electromagnetic MEMS-based microactuator which could be used as a switch for radio-frequency circuits. This microactuator based on a pot core–type electromagnet and a mover could be operated for more than 104 cycles. Its maximum switching distance is nearly 200 μm. Recently, planar coils, which are used to fabricate variable-focus liquid lenses, have been reported. Lee et al. [11] introduced a focal tunable liquid lens integrated with a planar electromagnetic actuator, and realized the integration of the actuator and lens body. However, in order to improve the zooming effect, they used an ultrathin polydimethylsiloxane (PDMS) film (about 11 μm) to seal the driven chamber and optical chamber, which requires a high level for fabrication technology [11]. In this paper, a novel electromagnetically actuated variable-focus liquid lens is presented. Based on ultraviolet photolithography (UV-LIGA) technology, a planar electromagnetic actuator, which consists of two contrary rotating copper coils, is designed and fabricated. It is fixed on a polymethyl methacrylate (PMMA) substrate and provides a driving force for the variable-focus microlens. The experimental results demonstrate that the focal length of proposed liquid lens can be precisely controlled by regulating the magnitude of the current passed through the planar coil. When the actuation current changes from −1 to 1.2 A, the applied voltage varies between −5.5 and 6.6 V, and the whole focal length of the proposed liquid lens ranges from −133 to −390 mm and from 389 to 61 mm. Compared with previous approaches, the improved liquid lens has several merits: integration of the actuator and lens body, a smaller volume, a fully closed structure, and suitability for planar integration.

## 2. Device Structure and Mechanism

**Figure 1:**
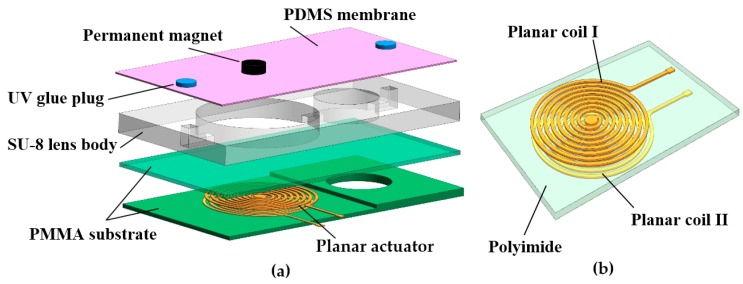
Schematic diagram of the variable-focus liquid lens: (**a**) 3D structure; (**b**) Structure of the planar electromagnetic actuator. PMMA: polymethyl methacrylate; PDMS: polydimethylsiloxane; UV: ultraviolet.

Figure 1 presents a schematic of the variable-focus liquid lens developed in the present study. As shown in Figure 1, this liquid lens consists of a PMMA protective structure containing two layers: a rectangular SU-8 middle layer containing two chambers with the same radius, namely the optical chamber and driven chamber which connect via a microchannel; a thin PDMS elastic membrane; a permanent magnet which attaches to the PDMS membrane above the driven chamber; a planar electromagnetic actuator which is made up of two contrary rotating copper coils and a polyimide insulating layer. A through-hole was fabricated on the polyimide layer to facilitate an electrical connection between the two copper coils. The detail parameters of the variable-focus liquid lens are shown in Table 1.

**Figure 2:**
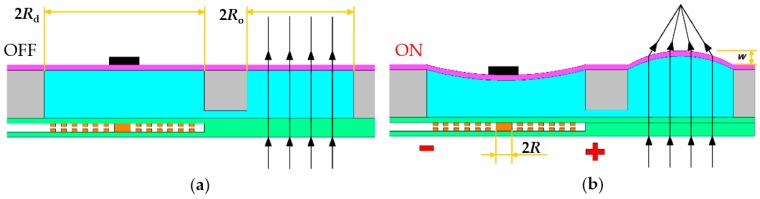
The operation mechanism of the proposed liquid lens: (**a**) Relaxed state; (**b**) Applied current to the liquid lens.

Figure 2 illustrates the working principle of the proposed variable-focus liquid lens. When current is applied to the planar coil, a magnetic force is induced between the planar electromagnetic actuator and the permanent magnet, causing the driven chamber PDMS membrane to deflect and create an actuation effect, similar to that observed for the lens reported by Yu et al. [21]. Following this, the membrane of the optical chamber is deformed in the opposite direction. At the relaxed state, the focal length tends to infinitely increase. As the current increases, the magnetic force increases gradually, the displacement values gradually grow larger, the change of the focal length is more obvious, and the purpose of changing the focal length is realized.

The magnitude of the driven force generated from the interplay between the planar electromagnetic actuator and the permanent magnet directly determines the performance of the variable-focus liquid lens. For the study of the magnetic field from the multi-turn planar coil when a current applied, it can be considered as the superposition of the magnetic field generated by a plurality of concentric current rings with different diameters. According to the Biot–Savart law, the magnetic field strength *H*(*z*) provided by the planar coil is given by:

(1) $$H\left(z\right)=\overset{n}{\underset{i=1}{\sum }}\frac{I{\left[R+\left(i-1\right)\tau \right]}^{2}}{{\left\{{\left[R+\left(i-1\right)\tau \right]}^{2}+z^{2}\right\}}^{\frac{3}{2}}}$$

where *z* is the distance between the permanent magnet and planar electromagnetic actuator, *I* is the current through the planar coil, *R* is the radius of the inner ring of the planar coil, τ is the spacing between adjacent coils. Referring to Equation (1), the magnetic force can be calculated by [25]:

(2) $$F_{z}=\int_{z}^{z+h}\frac{dH\left(z\right)}{dz}B_{r}Adz$$

where *F*_z_ is the magnetic force, *B*_r_ is the remanence of the permanent magnet material, *A* is the area of the permanent magnet bottom surface, *h* is the height of the magnet. According to the principle of hydrostatic transmission, the pressure exerted on the stationary liquid in a closed vessel can be transferred to any point in the connected liquid with the same size. So the force applied to the optical chamber membrane is equal to the magnetic force generated between the magnet and planar coil, and the relationship between the displacement values of the optical chamber membrane and *F*_z_ is given by [25]:

(3) $$\omega =\frac{F_{z}b^{2}}{64\pi D}$$

where ω is the displacement value, *b* is the radius of the optical chamber membrane, *D* = *EH*^3^/12(1 − θ^2^) is the flexural rigidity of the membrane, in which *E*, θ and *H* are the elastic modulus, Poisson’s ratio and the thickness of the PDMS membrane, respectively. So the focal length of this liquid lens can be described by:

(4) $$f=\frac{r}{n_{\text{liquid}}-1}$$

(5) $$r=\frac{b^{2}+\omega^{2}}{2\omega }$$

where *n_l_*_iquid_ is the refractive value of the liquid, and *r* is the radius of the optical chamber membrane. So as the magnetic force increases, the focal length has the tendency to be decreased, and the focal length can also be precisely controlled by the current applied to the planar coil.

## 3. Fabrication

The fabrication method of the proposed liquid lens is a three-stage procedure including planar electromagnetic actuator fabrication, lens body preparation and assembly of these two parts.

### 3.1. The Fabrication of the Planar Electromagnetic Actuator

**Figure 3:**
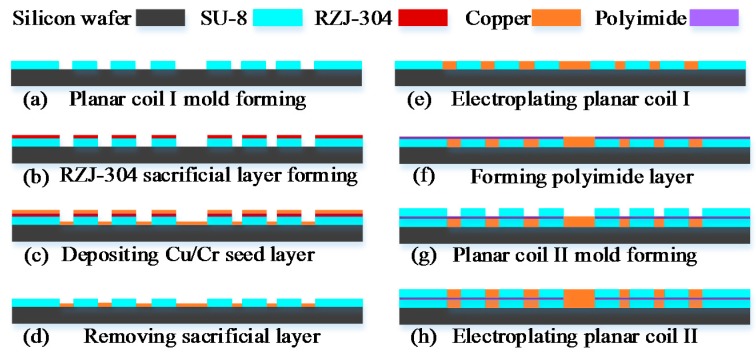
Schematic diagram of the planar electromagnetic actuator fabrication process. (**a**) Fabricate SU-8 mold for planar coil I; (**b**) spray RZJ-304 layer on the SU-8 substrate, and forming the sacrificial layer; (**c**,**d**) deposit Cu/Cr seed layer and pattern it by removing the sacrificial layer; (**e**) electroplating copper layer (~30 μm) on top of the seed layer within the mold; (**f**) deposit polyimide layer and pattern it; (**g**) fabricate SU-8 mold for planar coil II; (**h**) electroplating planar coil II within SU-8 mold.

A simple overview of the major steps adopted in the fabrication process of the planar electromagnetic actuator is presented in Figure 3. The step-by-step procedure used to fabricate the planar electromagnetic actuator can be summarized as follows:

The planar electromagnetic actuator was formed by spinning a photoresist layer (SU-8 2025, Micro Chem Corp., Newton, MA, USA) with a thickness of 30 μm onto a clean silicon wafer and cured on a well-leveled hot plate for 15 min at 65 °C and 30 min at 95 °C. Subsequently, a standard photolithography process was used to create a SU-8 mold for electrode I, as shown in Figure 3a. Then a positive photoresist layer (RZJ-304, Suzhou Ruihong Co., Suzhou, China) was sprayed on the substrate; after baking, photoetching, and development, the SU-8 layer was covered by the RZJ-304 layer and is shown in Figure 3b. A 50 nm chromium layer and 200 nm copper seed layer was then deposited on the substrate, and immersed in a remover agent for removing the RZJ-304 layer, as shown in Figure 3c,d. Then the planar coil I was formed by using an electroplating technology to deposit a copper layer with a thickness of 30 μm on top of the seed layer within the mold, as shown in Figure 3e. A photoimageable polyimide layer (ZKPI-510, POME Sci-tech Co., Beijing, China) with a thickness of 10 μm was spun on the planar coil I. Next, the polyimide layer undergoes photoetching and a developing process forming a via-hole to facilitate an electrical connection between planar coil I and planar coil II. Using an electroplating process, the via-hole was filled with a 5-μm-thick plug of copper, as shown in Figure 3f. A 30-μm-thick SU-8 2025 layer was coated onto the polyimide substrate using the spinning method and photopatterned to form a mold for the planar coil II, as shown in Figure 3g. Subsequently, the same method as that shown in Figure 3b,c was used to form the seed layer of the planar coil II. After sputter a 200-nm-thick copper seed layer on the mold, the planar coil II was obtained by using an electroplating process, as shown in Figure 3h. Then a polyimide coating resin layer (ZKPI-305, POME Sci-Tech Co.) was coated over the planar coil. Finally, in order to solidify the polyimide layer, this structure was baked at 150, 180, 250, and 300 °C for 1 h, respectively. The detail parameters of the micro-planar electromagnetic actuator are shown in Table 2.

### 3.2. The Fabrication of Lens Body

As Figure 1 shows, the lens body includes PDMS membrane, SU-8 substrate and PMMA protective structure. In this paper, the SU-8 substrate with a thickness of 1000 μm was formed through two standard photolithography process; the radius of the optical chamber is 5000 μm. The PMMA protective structure was fabricated by laser processing. The PDMS membranes used in the variable-focus liquid lens were fabricated using liquid PDMS (Sylgard 184, Dow Corning, Midland, MI, USA) prepolymer with mixing ratio of 10 (silicon resin):1 (silicone elastomer). The detail procedure used to form the PDMS membrane can be summarized as follows:

The fabrication process of PDMS membrane begins with cleaning a silicon wafer using an acetone solution and deionized (DI) water, after careful drying, a RZJ-304 photoresist layer around 2 μm thick was coated onto its surface by a spinning technique, after cured at 100 °C for 2 min, the PDMS prepolymer was spin-coated on the photoresist layer at the speed of 1200 r/min, cured it at a temperature of 80 °C about 30 min. Subsequently, the PDMS membrane was immersed in an acetone solution to remove the photoresist layer, after these steps, the PDMS membrane about 100 μm was obtained.

### 3.3. Assembly

**Figure 4:**
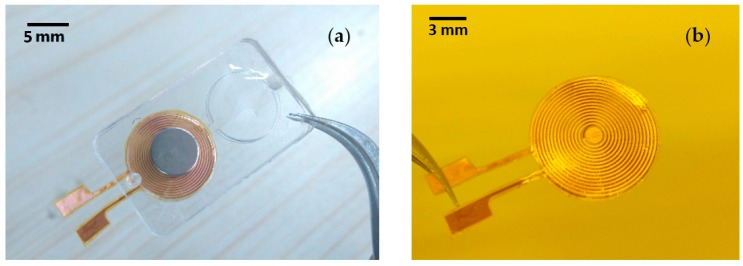
Prototype of the proposed liquid lens: (**a**) Sample of the lens; (**b**) Sample of the planar electromagnetic actuator (already removed the excess part of the polyimide layer).

Owing to the low surface energy of PDMS, it is difficult to create robust everlasting bonding between SU-8 substrate and PDMS membrane. Therefore, surface treatment is necessary to increase the surface energy of PDMS. In the assembling processes, we first using oxygen plasma-activated, and then immersed in a 0.5% Sodium Dodecyl Sulfate (SDS) solution to improving the surface energy of PDMS. After these steps, the PDMS membrane was bonded to the SU-8 substrate which also experienced oxygen plasma treatment. Subsequently, the SU-8 substrate, PMMA protective structure and the planar electromagnetic actuator were carefully aligned and bonded using UV glue (Loctite 3108, Henkel Co., Hartford, CT, USA). Deionized water as the filling liquid was injected to the enclosed spaces through the liquid inlet. The UV glue plugs were formed and used to choke both liquid inlet and outlet, forming a sealed environment. After these steps, a permanent magnet attachment process was executed, a small permanent magnet was attached to PDMS membrane upper surface using the same glue. The radius, thickness and remanence of the permanent magnet are 3.25 mm, 1.2 mm and 1.4 T, respectively. At last, the UV glue plugs were formed and used to choke both liquid inlet and outlet, forming a sealed environment. Figure 4 presents a photograph showing the fully-assembled liquid lens and the sample of the planar electromagnetic actuator. 

## 4. Experiment and Discussion

**Figure 5:**
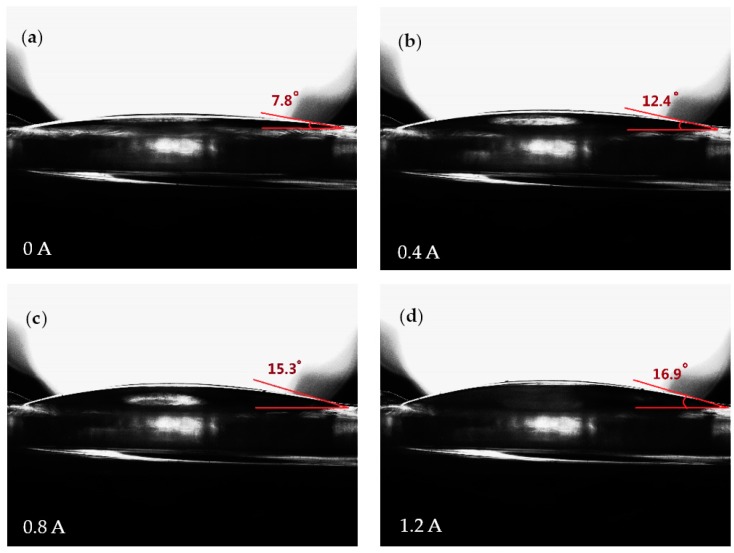
The shape change of the optical membrane with different applied currents. As the applied current increases, the PDMS membrane is pushed by the water and bulges up into a shape with a smaller radius of curvature.

Figure 5 shows the cross-section images of the optical chamber when the current applied varies between 0 and 1.2 A. The focal length change of the liquid lens is monitored by a direct current (DC) power supply. Figure 5 shows the initial stage when the planar electromagnetic actuator is not energized, and we can see that the shape of optical chamber membrane is not a plane due to the effect of gravity on the permanent magnet, the tangent angle was tested about 7.8°. When the current is applied, the resultant electromagnetic force applied on the magnet drags the membrane, and causes the PDMS membrane of the driven chamber to deflect as shown in Figure 5b–d. The tangent angle reached maximum of about 16.9° at *I* = 1.2 A.

**Figure 6:**
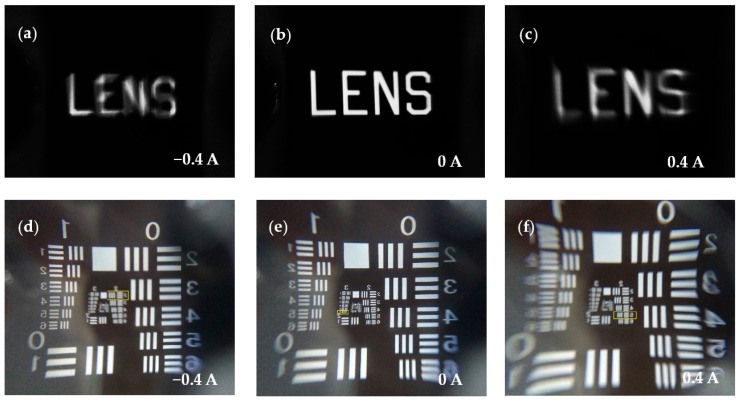
Imaging performance of the proposed variable-focus liquid lens at different applied currents: (**a**,**d**) *I* = −0.4 A; (**b**,**e**) *I* = 0 A; (**c**,**f**) *I* = 0.4 A.

To evaluate the liquid lens performance of imaging during the actuation process, we record the image of an object through the variable-focus liquid lens. The object is a chrome plate with capital letters saying “LENS”, which was kept 50 mm away from the optical chamber; thus, the object is always within the focal length of the liquid lens. Therefore, we can observe an upright virtual image. The camera records the images through the liquid lens at different currents from −1 to 1.2 A, and Figure 6 shows the images obtained at −0.4, 0, and 0.4 A, respectively. At the initial state, we record a mostly clear picture of the object, as shown in Figure 6b. The image changes from when we apply the current to the planar coil are shown in Figure 6a,c. When we remove the current, the PDMS membrane moves backwards to its original position automatically due to the high interface tension with the sidewall of the structure. The resolution of this liquid lens is tested by using the transparent film with a 1951 USAF resolution chart, and the test results are shown in Figure 6d–f. At the rest state, the liquid lens can identify smaller patterns in group 3, element 6, and the corresponding resolution of the liquid lens is about 14.3 lp/mm. In addition, when *I* = −0.4 A, it can resolve group 2, element 2, and in this case, the resolution is about 4.49 lp/mm. When the applied current changes to 0.4 A, the lens can resolve group 2, element 5, and the resolution is 6.35 lp/mm.

**Figure 7:**
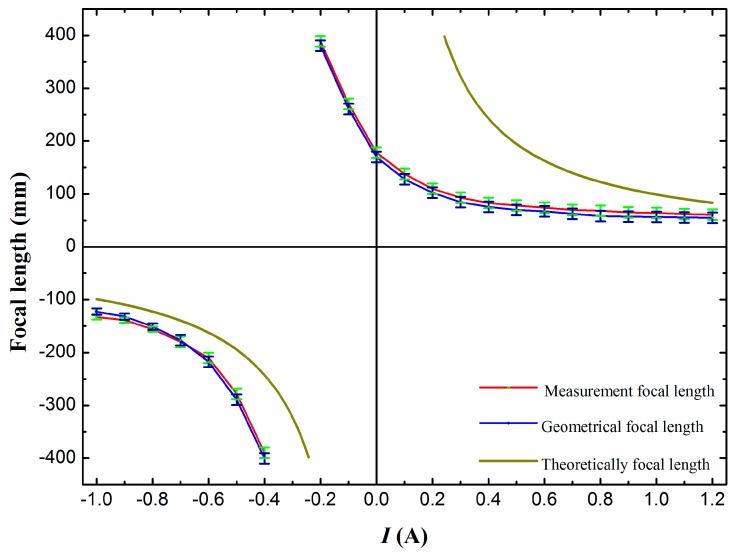
Change of focal length of the electromagnetically actuated variable-focus liquid lens with applied current.

For a variable-focus liquid lens, the key parameter is its capability for focal length tuning. Accordingly, in our experiment, in order to measure the focal length of this liquid lens, we use a He-Ne laser beam (*λ* = 632.8 nm), which has been expanded and collimated, to irradiate the liquid lens. A screen is placed below the liquid lens for testing the focal point. The laser beam passes through the liquid lens and focuses at a spot on the surface of the screen. The measured focal length versus the current change is shown in Figure 7 in red. With the applied current increased from −1 to −0.4 A, the focal length changes from −133 to −390 mm, and then with an increase of the current to 1.2 A, the focal length changes from 389 to 61 mm. In addition, the theoretically calculated focal length and the geometrical focal length are also demonstrated in Figure 7. From the results, we can see that the three kinds of focal lengths vary in a similar trend with the increasing current. The experimental measurement results and the geometrical focal length basically coincide. However, the results of the theoretical calculation and the two other results are not well matched. After several test analyses, we find the possible source of this phenomenon occurring is the effect of gravity on the permanent magnet. Before the magnet is placed on the PDMS membrane, both the optical chamber membrane and driven chamber membrane are all planar, and the measured focal length tends toward infinity. After fixing the magnet (about 6 g) on the driven chamber membrane, the driven chamber membrane generates a downward displacement due to the gravity of the magnet; following this, the membrane of the optical chamber is deformed in the opposite direction, and the displacement is tested at about 450 μm.

**Figure 8:**
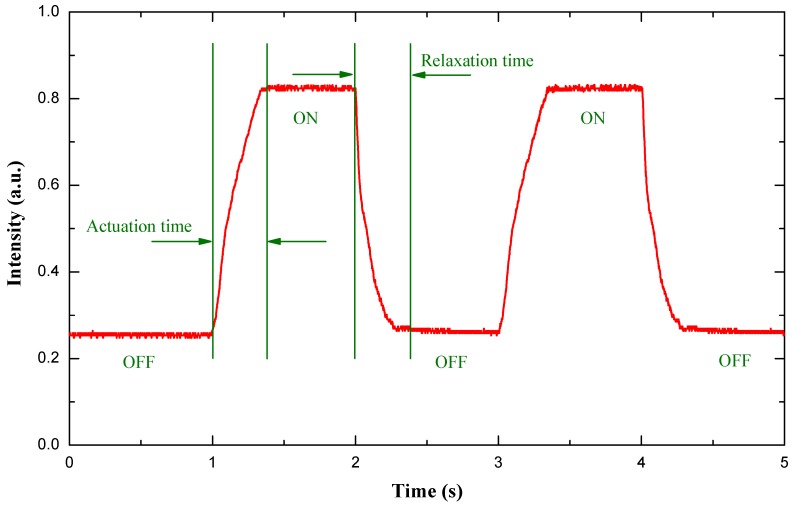
The measured response time of the proposed liquid lens with *I* = 0.8 A and λ = 632.8 nm.

When the liquid lens is used for dynamic imaging, the response time is an important parameter. To measure the response time, we set about characterizing this property using a similar method as that described in [19]. The measuring procedure and test result can be summarized as follows. The same He-Ne laser beam was used. A photodiode detector was placed below the optical chamber to receive the laser beam. The photo-sensitive area of the photodiode detector is smaller than the aperture of the liquid lens, so part of the laser beam was received by the detector when it was placed in a defocusing state. When a current was applied to the planar coil, the focusing point of the liquid lens shifted, and the focal length progressively grew smaller, so more light across the liquid lens was received by the detector. Figure 8 shows the time-dependent light intensity when a square-wave current signal was sent to the planar electromagnetic actuator. When the applied current equals 0.8 A, the measured result shows that the actuation time and relaxation time are 340 and 460 ms, respectively.

## 5. Conclusions

In this paper, a new variable-focus liquid lens actuated by a planar electromagnetic actuator is reported. It has several advantages, such as small size, suitability for planar integration, and so on. At first, the structure of the variable-focus liquid lens is demonstrated. It consists of a permanent magnet, a PDMS elastic membrane, a rectangular SU-8 middle layer, two PMMA protective structures and a planar electromagnetic actuator. Then the preparation process of the variable-focus liquid lens is also introduced. In the experiment, the focal length and curvature of the liquid lens turned out to be regulated easily by varying the applied current through the planar coil. The test results show that when the applied current is increased from −1 to 1.2 A, the liquid lens changes from a concave lens to a convex lens, and the transition current is about −0.3 A. The whole focal length of the proposed liquid lens ranges from −133 to −390 mm and from 389 to 61 mm. The theoretical focal length is also calculated. The measured focal length and the theoretical focal length vary in a similar trend with the increasing current, but when no current is applied, the measured focal length is not at infinity due to the effect of gravity on the permanent magnet, and the focal length is tested at about 178 mm. The response time of the liquid lens is measured. Then a square-wave current of 0.8 A is applied, and the response time is measured at about 340 ms. When the current is removed, the recovery time is around 460 ms. However, the problem of high energy consumption of the proposed liquid lens is found. In the future, the driving current and the dimension of the liquid lens can be further reduced by optimizing the parameters of the planar electromagnetic actuator and other components. The fabrication process can be improved to realize a more compact system. Based on the results obtained, the proposed liquid lens has potential applications such as medical detecting instruments, imaging systems and information displays.

## Figures and Tables

**Table 1:** Parameters of designed variable-focus liquid lens.

Parameters	Data
Length	28 mm
Width	18 mm
Thickness apart from the magnet	2.4 mm
Radius of driven chamber *R*_d_	6 mm
Radius of optical chamber *R*_o_	5 mm

**Table 2:** Parameters of designed micro-planar electromagnetic actuator.

Parameters	Data
Turns	15
Width	280 μm
Thickness	35 μm
Inner radius *R*	1000 μm
Spacing	140 μm
Resistance	5.5 Ω

## Supplementary Materials

The following are available online at www.mdpi.com/2072-666X/7/10/190/s1. The video of an object observed through the liquid lens when the current is applied to the planar coil.

## References

[1] Chiu C.P., Chiang T.J., Chen J.K., Chang F.C., Ko F.H., Chu C.W., Kuo S.W., Fan S.K. (2012). Liquid lenses and driving mechanisms: A Review. J. Adhes. Sci. Technol..

[2] Yu H.B., Zhou G.Y., Lee F.W., Wang S.H., Leung H.M. (2009). A liquid-filled tunable double-focus microlens. Opt. Express.

[3] Ren H., Fox D., Anderson P.A., Wu B., Wu S.T. (2006). Tunable-focus liquid lens controlled using a servo motor. Opt. Express.

[4] Dong L., Agarwal A.K., Beebe D.J., Jiang H. (2006). Adaptive liquid microlenses activated by stimuli-responsive hydrogels. Nature.

[5] Wei K., Domicone N.W., Zhao Y. (2014). Electroactive liquid lens driven by an annular membrane. Opt. Lett..

[6] Liu C., Wang Q.H., Yao L.X., Wang M.H. (2014). Adaptive liquid lens actuated by droplet movement. Micromachines.

[7] Li C., Jiang H. (2012). Electrowetting-driven variable-focus microlens on flexible surfaces. Appl. Phys. Lett..

[8] Ren H., Xianyu H., Xu S., Wu S.T. (2008). Adaptive dielectric liquid lens. Opt. Express.

[9] Chang J.H., Jung K.D., Lee E., Choi M., Lee S., Kim W. (2012). Varifocal liquid lens based on microelectrofluidic technology. Opt. Lett..

[10] Wang W., Fang J. (2006). Design, fabrication and testing of a micromachined integrated tunable microlens. J. Micromech. Microeng..

[11] Lee S.W., Lee S.S. (2007). Focal tunable liquid lens integrated with an electromagnetic actuator. Appl. Phys. Lett..

[12] Ren H., Fan Y.H., Wu S.T. (2004). Liquid-crystal microlens arrays using patterned polymer networks. Opt. Lett..

[13] Cheng C.C., Chang C.A., Yeh J.A. (2006). Variable focus dielectric liquid droplets lens. Opt. Express.

[14] Ren H., Fan Y.H., Lin Y.H., Wu S.T. (2005). Tunable-focus microlens arrays using nanosized polymerdispersed liquid crystal droplets. Opt. Commun..

[15] Li C., Jiang H. (2014). Fabrication and characterization of flexible electrowetting on dielectrics (EWOD) microlens. Micromachines.

[16] Li X., Tian H., Shao J., Ding Y., Chen X., Wang L., Lu B. (2016). Decreasing the saturated contact angle in electrowetting-on-dielectrics by controlling the charge trapping at liquid–solid interfaces. Adv. Funct. Mater..

[17] Li L., Liu C., Ren H., Deng H., Wang Q.H. (2015). Annular folded electrowetting liquid lens. Opt. Lett..

[18] Xu M., Xu D., Ren H., Yoo I.S., Wang Q.H. (2014). An adaptive liquid lens with radial interdigitated electrode. J. Opt..

[19] Jin B., Xu M., Ren H., Wang Q.H. (2014). An adaptive liquid lens with a reciprocating movement in a cylindrical hole. Opt. Express.

[20] Xu M., Wang X., Ren H. (2015). Tunable focus liquid lens withradial-patterned electrode. Micromachines.

[21] Yu H., Zhou G., Chau F.S., Sinha S.K. (2011). Tunable electromagnetically actuated liquid-filled lens. Sens. Actuators A Phys..

[22] Thielicke E., Obermeier E. (2000). Microactuators and their technologies. Mechatronics.

[23] Lee C.Y., Chen Z.H., Chang H.T., Wen C.Y., Cheng C.H. (2009). Design and fabrication of novel micro electromagnetic actuator. Microsyst. Technol..

[24] Hartley A.C., Miles R.E., Corda J., Dimitrakopoulos N. (2008). Large throw magnetic microactuator. Mechatronics.

[25] Chang H.T., Lee C.Y., Wen C.Y. (2007). Design and modeling of electromagnetic actuator in MEMS-based valveless impedance pump. Microsyst. Technol..

